# Getting in: The structural biology of malaria invasion

**DOI:** 10.1371/journal.ppat.1007943

**Published:** 2019-09-05

**Authors:** Hirdesh Kumar, Niraj H. Tolia

**Affiliations:** Laboratory of Malaria Immunology and Vaccinology, National Institute of Allergy and Infectious Diseases, National Institutes of Health, Bethesda, Maryland, United States of America; Children's Hospital of Philadelphia, UNITED STATES

Malaria affects one third of the world’s population and kills hundreds of thousands across the globe annually. Among the species that cause malaria in humans, *Plasmodium falciparum (Pf)* and *P*. *vivax (Pv)* are the most virulent and responsible for the majority of the mortality and morbidity attributed to malaria.

Malaria infection begins when an infected female *Anopheles* mosquito bites an individual and releases *Plasmodium* sporozoites, the motile and infective form of the parasite, into the skin. The deposited sporozoites migrate through the skin, enter the circulatory system, and traffic to the liver. This migration requires traversal of the sporozoite through diverse cell types of distinct host tissues. Inside the liver, *Plasmodium* sporozoites first undergo mandatory, asymptomatic, acyclic intrahepatic development before mature merozoites egress and infect red blood cells. The cyclic blood stage involves repeated rounds of invasion and replication within red blood cells and is responsible for all the disease symptoms and complications. During the blood stage, some parasites develop into gametocytes, which are the first step in the sexual cycle. The *Plasmodium* life cycle continues as a mosquito feeds on an infected host and takes up gametocytes in a blood meal. The parasites undergo sexual reproduction in the mosquito, which results in mature sporozoites that then propagate the next cycle of infection.

*Plasmodium* is predominantly intracellular during blood stage growth, and this protects parasites from the host immune response. However, the parasite is vulnerable when it is extracellular during traversal and prior to host cell invasion. Components of the *Plasmodium* invasion machinery are pathogen-specific and surface-exposed, making them potential vaccine and/or drug targets that can be exploited to design therapeutics against the deadly parasite. Structural studies provide the precise definition of the linear and nonlinear conformational neutralizing epitopes that can be exploited to improve the immunogen design of malaria vaccines. In addition, the structural details of *Plasmodium* specific ligands and ligand/receptor complexes provide unprecedented insight into the mechanisms of interaction, invasion, and inhibition at the host–parasite interface.

In this review, we describe the structural and functional details of *Plasmodium*-specific invasion proteins involved in 1) traversal, 2) hepatocyte invasion, and 3) erythrocyte invasion (see **Fig 1**for summary). The current review focusses on the most virulent human malaria parasites, *P*. *falciparum* and *P*. *vivax*. Additional components of the invasion machinery are also excluded from the current study as they have been extensively reviewed elsewhere.

**Fig 1:**
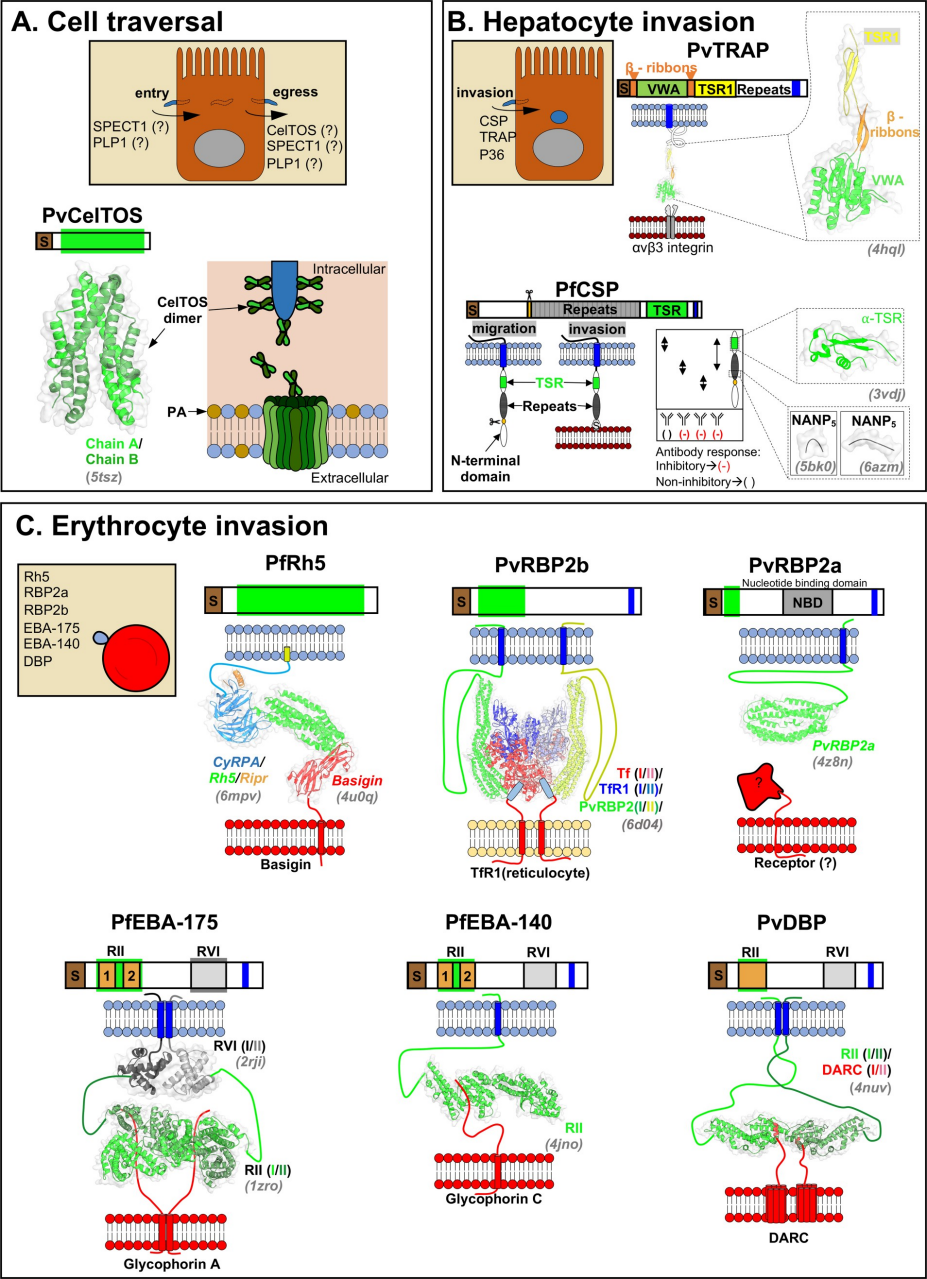
Structural and functional aspects of *Plasmodium* invasion proteins. Each section contains schematic representations of known domains, known mechanisms of actions, solved structures (in grey italic font), and known interacting host receptors. (A) CelTOS, PLP1, and SPECT1 are involved in the cell traversal, a process during which malaria parasites enter in, pass through, and finally exit from host cells. The mechanism of action of CelTOS is known, while SPECT1 and PLP1 are understudied. CelTOS forms pore at the inner leaflet of host cell membranes. (B) CSP, TRAP, and P36 are involved in hepatocyte invasion and initiate the mandatory intrahepatic development of the malaria parasite. While structural information for CSP and TRAP have been reported, the structural and functional details of P36 are unknown. The neutralizing (-) and non-neutralizing () epitope regions are highlighted in CSP cartoon. (C) Major *Plasmodium* proteins that are involved in the erythrocyte invasion are shown. PfRh5, PvRBP2a, and PvRBP2b are members of reticulocyte binding family. PfEBA-175, PfEBA-140, and PvDBP contain a conserved host receptor–binding domain, region II (RII), and are members of the erythrocyte binding-like family. Please note that the roman numbers within brackets represent different chain IDs in the crystal structures. CeITOS, cell traversal protein for ookinetes and sporozoites; CSP, circumsporozoite protein; PfEBA, *Plasmodium falciparum* erythrocyte binding antigen; PfRH, *Plasmodium falciparum* reticulocyte-binding homologue; PLP, perforin-like protein; PSPECT1, sporozoite protein essential for cell traversal 1; PvRBP, *Plasmodium vivax* reticulocyte-binding proteins; TRAP, thrombospondin-related adhesive protein.

## Traversal precedes invasion

Sporozoites are deposited into the dermis of the host upon the bite of an infected mosquito. These motile sporozoites traverse through the skin to find blood vessels and subsequently reach the liver through the circulatory system. Before a sporozoite can invade and replicate in a hepatocyte, it must traverse several physical barriers, including fibroblasts, Kupffer cells, and sinusoidal endothelial cells, to reach the target hepatocyte. Host cell traversal is the process of parasite entry into, passage through, and egress from host cells without lysis. Host cell traversal protects the vulnerable sporozoite from phagocytosis, primes the sporozoite through the activation of apical exocytosis, and prepares the motile sporozoite for invasion [1]. In addition, the release of hepatocyte growth factor during sporozoite traversal enhances the infection rate of neighboring cells. *Plasmodium* uses stage-specific pore-forming proteins to disrupt host cell membranes to either enter or exit host cells during traversal, and to egress from the parasite-built parasitophorus membrane after invasion and replication. The characterized pore-forming proteins include sporozoite protein essential for cell traversal 1 (SPECT1), perforin-like proteins (PLPs), and cell traversal protein for ookinetes and sporozoites (CelTOS) (**Fig 1A**).

SPECT1 and PLP1 are essential *Plasmodium* proteins that may have possible roles in cell traversal. The targeted disruption of *P*. *falciparum* SPECT1 or PLP1 cause reduced infectivity of sporozoites in liver-stage development in humanized mice [2]. However, mechanisms of cell traversal of these two proteins are yet to be defined.

Cell traversal protein for ookinetes and sporozoites (CelTOS) is a unique *Plasmodium* pore-forming protein that is required for cell traversal in both the mammalian host and the mosquito vector. Recently, the crystal structure of *P*. *vivax* CelTOS revealed an all α-helical, tuning fork–shaped dimer structure that resembled membrane-disrupting proteins from viruses and bacteria (**Fig 1A**) [3]. *P*. *vivax* and *P*. *falciparum* CelTOS can bind phosphatidic acid (PA), an inner leaflet abundant lipid, suggesting a plausible inside-out function of this protein during parasite traversal (**Fig 1A**). CelTOS forms pores in liposomes containing PA as observed by negative stain transmission electron microscopy. The protected hydrophobic core of this soluble dimer CelTOS structure suggests that a significant conformational rearrangement is mandatory to form a pore in cell membranes. Further work is needed to explore the conformation of the lipid-bound CelTOS. Antibodies raised against *P*. *falciparum* CelTOS protect from infection, and thus, CelTOS is a vaccine candidate being evaluated currently in clinical trials [4].

## Productive hepatocyte invasion

Following initial cell traversal, migratory sporozoites establish hepatocyte infection and undergo exponential growth to develop tens of thousands of primary merozoites. During active invasion, sporozoite surface proteins interact with host receptors to facilitate entry into the host cell. The three most actively studied sporozoite surface coat proteins are circumsporozoite protein (CSP), thrombospondin-related adhesive protein (TRAP), and P36 (**Fig 1B**).

CSP is the most abundant surface protein on sporozoites and has multiple roles in sporozoite development, gliding motility, and active invasion. CSP is composed of a central repeat region (approximately NANP_25-49_) which is diverse among different *Plasmodium* species. This repeat region is flanked by conserved N- and C-terminal domains. The N-terminus of CSP contains a charged protease-cleavage site known as region I (RI). The C-terminus contains a short, conserved sequence in region III (RIII) and a known thrombospondin-like type 1 repeat (TSR1) cell adhesive motif (**Fig 1B**). Antibodies against PfCSP C-terminal, induced by live sporozoite vaccination in humans, are ineffective against the malaria infection [5]. On the contrary, the antibodies against the central repeat region and the junction (between the N-terminus and the central repeat region) protect mice and mosquitoes from infection [6–8]. In these particular studies, the protective antibodies preserve the germline encoded residues within the paratope, suggesting that the naïve human B cell repertoire possesses the prerequisite for anti-CSP immunity that are further improved through somatic hypermutations [7, 8]. The CSP repeats also facilitate direct homotopic interactions between two monoclonal antibodies isolated from humans with repeated malarial infection [9]. These homotypic antibody interactions appear to be strongly selected through affinity maturation [9].

The individual CSP domains function in a stage- and time-specific manner. RI recognizes heparan sulfate proteoglycans (HSPG) on the salivary gland in the mosquito vector [10], and this domain is proteolytically cleaved, exposing the TSR-domain to interact with the highly sulfated HSPGs on hepatocytes (**Fig 1B**) [11]. The crystal structure of RIII region and TSR domain of *P*. *falciparum* CSP revealed a unique αTSR domain [12], where the amphipathic α-helix of RIII region runs orthogonal to the classic TSR homology region creating a hydrophobic pocket, which is conserved in different *Plasmodium* species (**Fig 1B)**. This pocket is proposed to have a possible role in host interaction. The central repeat region (NANP_18_) and αTSR domain are components of the leading malaria vaccine RTS,S/AS01 [12]. RTS,S/AS01 lacks the junctional residues, which were recently shown to be targets of the protecting antibodies [6–8]. In addition to the central repeat (NANP_18_), highly neutralizing anti-CSP antibodies engage the junctional residues between the N-terminal domain and repeat region [8], suggesting that these residues need to be considered in the structure-based design of the next generation CSP malaria vaccine.

TRAP also contains a TSR domain and is involved in host invasion and gliding motility. TRAP localizes to the plasma membrane and translocates from the anterior to the posterior end of the sporozoite during invasion. In TRAP, the N-terminus encompasses a rigid domain that shows structural homology to the von Willebrand factor (vWF) type A-domain (**Fig 1B)** [13]. This vWF domain is followed by the flexible TSR domain. At the C-terminus, TRAP contains a conserved cytoplasmic tail that interacts with the gliding motor of the parasite. TRAP is thought to function via a stick-and-slip model [13]. When a substrate binds to the metal-ion-dependent-adhesive-site (MIDAS) of the vWF domain, the vWF domain becomes activated and assists in the formation of extensible β-ribbons between vWF and TSR domains that link the invading sporozoite surface to its gliding motor, an apicomplexa specific, actomyosin-based locomotory system (**Fig 1B)** [14]. In *Plasmodium*, stage-specific TRAP members exist that are essential for the invasion of the merozoite (MTRAP), ookinete (CTRAP), or salivary gland sporozoites (TRAP) [14]. Recently, integrin αvβ3, a heterodimer of integrin alpha v and integrin beta 3, was identified as the host receptor for *P*. *falciparum* TRAP using a systematic extracellular protein screening approach [15]. An inactivated adenovirus-based multiple epitope TRAP (ME-TRAP) vaccine is shown to protect animals and humans against infection [16].

P36 is a 6-cysteine domain containing protein. Recent genetic analyses proposed a role for P36 in liver-stage invasion [17, 18]. Interestingly, P36 from *P*. *falciparum* binds the host receptor CD81, while *P*. *vivax* P36 binds to scavenger receptor BI (SR-B1) as the hepatocyte receptor during effective infection [18]. *Plasmodium* parasites contain fourteen 6-cysteine domains containing proteins, which are conserved among different *Plasmodium* species and play crucial role in fertilization, parasitophorous vacuole membrane fitness, and immune evasion [19]. Therefore, several members of this family are potential vaccine candidates [20].

## Invading red blood cells

Primary merozoites released from ruptured hepatocytes enter into the blood stream, invade erythrocytes, and develop into ring, trophozoite, and schizont stages, culminating in the formation of 16 to 32 mature merozoites. Each of these merozoites can invade a fresh erythrocyte and continue the cyclic, asexual blood stage development. Malaria parasites also exhibit distinct red cell tropism with *P*. *falciparum* invading reticulocytes as well as mature erythrocytes, while *P*. *vivax* is specific for reticulocytes.

Unlike all other stages, the fluidic nature of blood stage infection subsides the requirement of early traversal but complicates the invasion process. Red cell invasion by the parasite involves 1) initial interactions causing erythrocyte deformation, 2) apical interactions and invasion, and 3) a final recovery phase. This review covers the apical invasion and the parasite ligands involved therein (**Fig 1C**). Reticulocyte-binding ligand (RBL) and erythrocyte-binding like (EBL) are two critical protein families involved in red blood cell invasion (**Fig 1C**).

*P*. *falciparum* reticulocyte-binding homologue (PfRh) is the reticulocyte binding family that consists of PfRh1, PfRh2a, PfRh2b, PfRh4, and PfRh5. Receptors have been identified for PfRh4 (complement receptor 1) [21] and PfRh5 (basigin) [22]. While antibodies raised against each of these proteins inhibit parasite growth, gene knockout studies suggest that except for PfRh5, all Rh proteins have redundant functions and are nonessential for parasite survival [23].

PfRh5 is a leading blood stage vaccine candidate and an exceptional member of the Rh family as antibodies that prevent the interaction of PfRH5 with the host receptor basigin neutralize diverse lab and field isolates of *P*. *falciparum* [22]. The PfRh5 structure consists of a novel fold in which two bundles of 3-helices come together and form the binding site for the receptor basigin (**Fig 1C**) [24]. Rh5 forms a complex with CyRPA and RIPR during invasion and a recently solved cryo-electron microscopy structure suggests that the ternary complex, Rh5-CyRPA-Ripr, positions parallel to the erythrocyte membrane before Rh5 and Ripr rearrange and incorporate into the erythrocyte cell membrane [25, 26]. Alternately, the N-terminus of Rh5 has also been proposed to interact with P113, resulting in a distinct complex that is capable of binding basigin [27]. Rh5 lacks a transmembrane domain and tethers to the merozoite surface through interaction with a glycosylphosphatidylinositol (GPI)-linked protein such as P113 and/or CyRPA [27, 28]. Divergent roles for the multiple PfRh5 complexes have been proposed, with functions in parasite attachment and anchoring [27] and membrane insertion and pore-formation [25], however, further experiments need to be performed to confirm the roles of Rh5-complexes.

*P*. *vivax* reticulocyte-binding proteins (PvRBPs) are the homologs of PfRhs, and this family is composed of 11 proteins [29].

PvRBP2b was recently shown to bind transferrin receptor 1 (TfR1), a highly expressed surface receptor on a variety of mammalian tissues, including reticulocytes, that delivers iron-loaded transferrin (Tf) glycoprotein into cells to maintain iron homeostasis [30]. TfR1 is lost during the reticulocyte maturation process and therefore absent on the surface on RBCs. This possibly explains the reticulocyte-specific invasion of *P*. *vivax*, although maturation of DARC has also been proposed [31]. A recently solved cryo-electron microscopy structure of the *P*. *vivax* RBP2b:TfR1:Tf ternary complex reveals how *P*. *vivax* uses *Pv*RBP2b to hijack host TfR1 to invade the host reticulocyte without affecting binding of TfR1 to Tf [32]. PvRBP2b interacts with TfR1 and Tf through three principal sites. These include the apical domain and the protease-like domain of TfR1 and the N-terminal region of Tf (**Fig 1C**). Monoclonal antibodies raised against PvRBP2b prevent reticulocyte binding and reduce *P*. *vivax* invasion [30].

PvRBP2a is another structural homolog of PfRh5 in *P*. *vivax* and consists of a conserved kite-shaped domain but possesses distinct surface properties, suggesting a recognition site for the alternate receptor (**Fig 1C**) [33]. In contrast to PfRh5, PvRBP2b and PvRBP2a are highly polymorphic, and this variation will have to be accounted for in future vaccine designs based on PvRBP2a.

EBL-family proteins are a second set of redundant invasion ligands that contain a conserved domain architecture including a conserved, *Plasmodium*-specific, host receptor–binding domain, region II (RII). In EBL-proteins, RII contains either a single or double Duffy Binding–like (DBL) domain. EBL-family proteins interact with the host receptor through the RII region. In *P*. *falciparum*, four EBL-proteins have been identified: PfEBA-175, PfEBA-140, PfEBA-181, and PfEBL-1. *P*. *vivax* has a single member known as Duffy binding protein (PvDBP).

PfEBA-175 interacts with glycophorin A (GpA) on the erythrocyte surface in a sialic acid-dependent manner. The crystal structure of RII in complex with ⍺-2,3-sialyllactose revealed that RII is a dimer and that the sialic acid binding sites are located at the dimer interface (**Fig 1C**) [34]. Site-directed mutagenesis of PfEBA-175 residues at the sialic acid binding sites impairs the ability of PfEBA-175 to bind erythrocytes [34], suggesting that the sialic acid binding site in the PfEBA-175 crystal structure is likely the glycan binding site used in GpA binding on the erythrocyte surface. Between the two DBL-domains of RII (F1 and F2), the F2 domain makes most of the contacts with the glycans in the complex structure, suggesting a greater role for the F2 in red cell invasion through PfEBA-175. Interaction studies using the full-length ectodomain of PfEBA-175 and glycosylated GpA suggested dimerization of PfEBA-175 is important for tight-binding to GpA and regions outside the RII domain of PfEBA-175 also contribute to GpA binding [35]. The protein backbone of GpA also contributes to binding, presumably by correctly presenting the multiple glycosylation sites for interaction [36, 37]. A PfEBA-175 specific antibody that inhibits parasite growth binds at the PfEBA-175 interface and engages the GpA binding residues and the dimer interface [38]. EBA-175 is shed post-invasion, and this protein clusters RBCs to facilitate rapid transfer of replicated parasites to new RBC hosts. Clustering also enables immune evasion from neutralizing antibodies that target the invasion machinery [39].

PfEBA-140 is another *P*. *falciparum* DBL-domain–containing protein that binds to glycophorin C (GpC) in a sialic-acid–dependent manner. The structure of PfEBA-140 RII domain, consisting of two DBL domains, revealed two sialic acid binding sites within a monomer of RII, each of which were contained within its respective DBL domain (**Fig 1C**) [40]. Strikingly, mutation of residues in the F1-sialic acid binding site abrogates binding to erythrocytes, in contrast to mutations in the F2-sialic acid binding site that had no effect [40]. This suggests the primary receptor binding site is in the F1 domain of PfEBA-140. The available structures of PfEBA-140 are all monomeric, and further work is needed to determine if receptor-bound oligomeric states exist. Antibodies raised against PfEBA-140 inhibit the invasion of multiple *P*. *falciparum* lab strains, suggesting the role of this ligand in parasite invasion, with antibodies that targeting F1 having a greater neutralizing potential [41, 42].

PfEBA-181 and PfEBL-1 are two other DBL domains containing proteins that have conserved EBL-family architecture but are not yet structurally characterized.

PvDBP is the functional ortholog of PfEBA175 in *P*. *vivax*. PvDBP binds the Duffy antigen receptor for chemokines (DARC) on the reticulocyte surface. Unlike PfEBA-175, the RII domain of PvDBP consists of a single DBL domain (**Fig 1C**). In vitro structural and biophysical studies of PvDBP RII identified a conserved stoichiometry of 2:2 between PvDBP and the host receptor DARC [43]. The sulphation of DARC Tyr41 increases binding of DBP to DARC, suggesting that post-translational sulphation of DARC plays a role in parasite invasion [44]. The structure of PkDBP identified a potential sulfo-tyrosine binding pocket that [45] is distinct from the DARC binding site and the dimer interface [43]. The receptor-binding pocket of PvDBP has limited polymorphisms in contrast to the other segments that are highly polymorphic [46]. Therefore, it is possible to develop therapeutics targeting the *P*. *vivax* DBP-DARC interface to reduce malaria. Although PvDBP is polymorphic, broadly neutralizing epitopes have been identified in the DBL domain [47]. Interestingly, DARC is also present on the erythrocyte surface, but the enhanced exposure of PvDBP binding pocket on young reticulocytes explains the *P*. *vivax* tropism [31].

In conclusion, through advanced tools in genetics, structure biology, and immunoparasitology, we have gained immense knowledge about the *Plasmodium* invasion machinery and its individual components in the last few decades. Future research will leverage the available structural information and explore the conformational space of the invasion machinery to design, develop, and optimize novel therapeutics. The preliminary success of RTS,S/AS01, the first marketed malaria vaccine, supports the idea that an infection-blocking malaria vaccine is indeed feasible. Understanding the mechanisms of *Plasmodium* invasion will guide development of novel vaccines to interrupt the invasion process and prevent disease and transmission of malaria.
